# Odd-parity magnetoresistance in pyrochlore iridate thin films with broken time-reversal symmetry

**DOI:** 10.1038/srep09711

**Published:** 2015-05-11

**Authors:** T. C. Fujita, Y. Kozuka, M. Uchida, A. Tsukazaki, T. Arima, M. Kawasaki

**Affiliations:** 1 Department of Applied Physics and Quantum-Phase Electronics Center (QPEC), University of Tokyo, Tokyo 113-8656, Japan; 2 Institute for Materials Research, Tohoku University, Sendai 980-8577, Japan; 3 PRESTO, Japan Science and Technology Agency (JST), Tokyo 102-0075, Japan; 4 Department of Advanced Materials Science, University of Tokyo, Kashiwa 277-8561, Japan; 5 RIKEN Center for Emergent Matter Science (CEMS), Wako 351-0198, Japan

## Abstract

A new class of materials termed topological insulators have been intensively investigated due to their unique Dirac surface state carrying dissipationless edge spin currents. Recently, it has been theoretically proposed that the three dimensional analogue of this type of band structure, the *Weyl Semimetal* phase, is materialized in pyrochlore oxides with strong spin-orbit coupling, accompanied by all-in-all-out spin ordering. Here, we report on the fabrication and magnetotransport of Eu_2_Ir_2_O_7_ single crystalline thin films. We reveal that one of the two degenerate all-in-all-out domain structures, which are connected by time-reversal operation, can be selectively formed by the polarity of the cooling magnetic field. Once formed, the domain is robust against an oppositely polarised magnetic field, as evidenced by an unusual odd field dependent term in the magnetoresistance and an anomalous term in the Hall resistance. Our findings pave the way for exploring the predicted novel quantum transport phenomenon at the surfaces/interfaces or magnetic domain walls of pyrochlore iridates.

A zero-gap surface state protected by time-reversal symmetry is formed at the surface of topological insulators, where electrons with up or down spins counter-propagate^1^,^2^,^3^,^4^,^5^,^6^. When a topological insulator is doped with magnetic elements^5^,^6^,^7^, these surface states may become gapped, resulting in the dissipationless flow of a single chiral edge mode which effectively exhibits the *ν* = 1 quantum Hall state in the absence of an external magnetic field^8^,^9^. A similar zero-gap state in three dimensional momentum space, referred to as Weyl Semimetal (WSM) phase^10^,^11^,^12^,^13^, has recently been predicted to emerge in a certain class of pyrochlore oxide with broken time-reversal symmetry^14^,^15^. As WSM possesses a topologically non-trivial band structures, provided momentum is conserved, a unique surface state accompanied with edge currents is predicted at interfaces with trivial insulators as in the case of topological insulators^15^,^16^,^17^.

Pyrochlore oxides, formulated as *A*_2_*B*_2_O_7_, are comprised of cation sublattices forming a corner-sharing tetrahedron network (Fig. 1(a); Ref. 18) where antiferromagnetically coupled spins at the cation sites cannot determine their configurations uniquely due to geometrical frustration. In the case of lanthanides (*Ln*) iridate pyrochlore (*Ln*_2_Ir_2_O_7_; *A* = *Ln*, *B* = Ir), Dzyaloshinski-Moriya interaction enhanced by strong spin-orbit coupling can stabilize an all-in-all-out antiferromagnetic spin order for each cation sublattice^15^,^19^,^20^, in which tetrahedra with all four spins at the vertices pointing inward or outward are alternatingly arranged along <111> direction, as shown in Fig. 1(b). A notable feature of this spin structure is while characterised as antiferromagnetic, two distinct spin arrangements exist, of which are interchangeable by time-reversal operation. We hereafter refer to these two spin arrangements as A-domain and B-domain. This characteristic antiferromagnetic ordering has begun to attract attention as a key ingredient to substantiate WSM and to explore the surface states at the boundaries between A- and B-domains^21^.

To investigate such intriguing phenomena through electrical measurements, it is necessary to control the magnetic domains and the carrier density of a single crystal. A promising approach to realising this is the growth of thin films, as it may enable the control of artificial magnetic domain boundaries and the exploration of carrier transport at such interfaces, as well as tunability of the Fermi energy at Weyl point by pursuing field-effect transistor devices. Nevertheless, obtaining high-quality *Ln*_2_Ir_2_O_7_ single crystal has remained challenging even in bulk because of the low reactivity of metallic iridium and the volatility of iridium peroxides^22^. Here, we report the fabrication of single crystal pyrochlore iridate thin films, Eu_2_Ir_2_O_7_ (*Ln* = Eu), and their magnetotransport properties mediated by all-in-all-out spin ordering. We chose *Ln* = Eu because of the total magnetic moment *J* = 0 of Eu^3+^ for among *Ln*_2_Ir_2_O_7_ series, this compound is a sole and ideal platform for studying carrier transport in the background of all-in-all-out spin structure composed of the Ir^4+^ moment in *J*_eff_ = 1/2 state^23^. The results indicate that the magnetic domain structure is detected via peculiar asymmetric term in the magnetoresistance (MR) and zero-field offset in Hall resistance. These observations provide controllability of the exotic electronic phase in this compound towards accessing WSM.

## Results

### Sample fabrication and structural property

**Figure 1:**
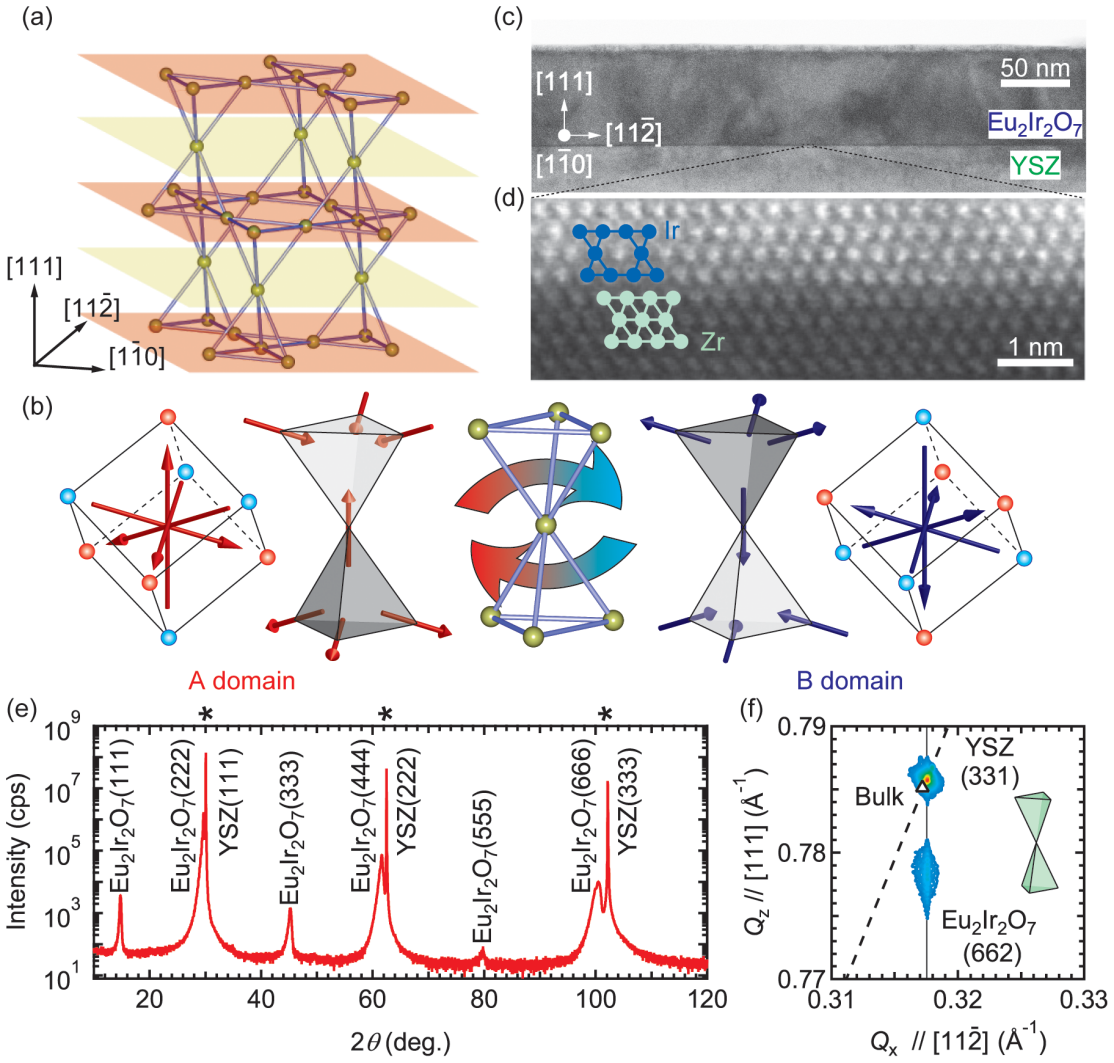
Crystal and spin structures of the pyrochlore lattice. (a) The Ir sublattice of Eu_2_Ir_2_O_7_. Kagome and triangular lattices formed by Ir are located at orange and yellow planes, respectively. (b) Two distinct all-in-all-out spin structures, named as A-domain and B-domain, in the pyrochlore lattice. When four spins at tetrahedral vertices are consolidated at the centre of cube, they represent a magnetic octupole indicated by blue and red spheres. (c) Phase contrast image of high-resolution TEM of a Eu_2_Ir_2_O_7_ (111) film on a YSZ (111) substrate. (d) Atomically resolved HAADF-STEM image at the Eu_2_Ir_2_O_7_/YSZ interface. Triangular cross-sectional lattices composed of Ir and Zr are schematically shown. (e) *θ*-2*θ* scan of X-ray diffraction. Peaks from the substrate are marked with asterisks. (f) The reciprocal space mapping around the YSZ (331) peak. The peak position of bulk Eu_2_Ir_2_O_7_ is indicated by an open triangle. The solid line indicates that Eu_2_Ir_2_O_7_ is coherently grown on the substrate and expanded along [111] direction by 0.7%, which is illustrated by the pair of tetrahedra. An ideal pyrochlore lattice with cubic symmetry obeys the theoretical curve indicated by the dashed line.

In this study (111)-oriented Eu_2_Ir_2_O_7_ thin films were epitaxially grown on Y-stabilized ZrO_2_ (YSZ) (111) single crystal substrates by pulsed laser deposition (see Methods and Supplementary Fig. S1). Transmission electron microscopy (Figs. 1(c) and 1(d)) confirms the formation of a single-crystal Eu_2_Ir_2_O_7_ film free from crystalline domain boundaries. The macroscopic crystal structure was examined by X-ray diffraction (XRD), with the high quality of the films supported by the observation of a typical rocking curve of 0.09° for the Eu_2_Ir_2_O_7_ (222) peak (Fig. 1(e) and Supplementary Fig. S2). In Fig. 1(f), the reciprocal space mapping is shown, indicating that the lattice of the film is elongated along [111] direction by 0.7% with respect to in-plane lattices. The surface morphology was measured by atomic force microscope, which showed root mean square roughness of ~1 nm before and after post-growth annealing (Supplementary Fig. S3). The film thickness is fixed at about 70 nm.

### Longitudinal resistivity

**Figure 2:**
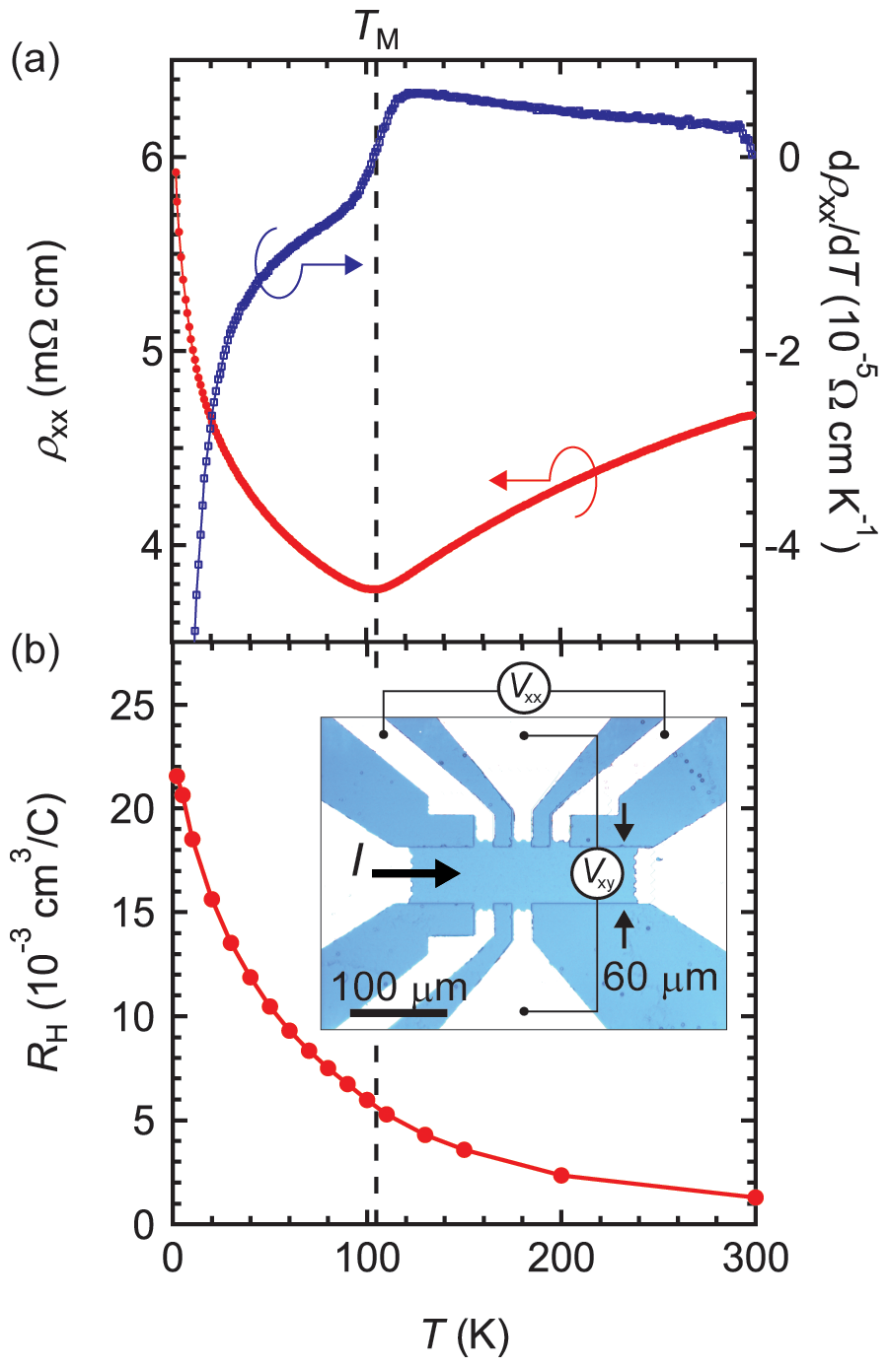
Temperature dependence of the longitudinal resistivity *ρ*_xx_ and Hall coefficient *R*_H_. (a) Temperature dependence of the longitudinal resistivity *ρ*_xx_ (left axis) and its temperature derivative (right axis). Magnetic transition temperature *T*_M_ is estimated at the minimum of the resistivity and 105 K. (b) Temperature dependence of the Hall coefficient *R*_H_. The inset shows an optical microscope image of the sample and the transport measurements configuration.

Figure 2(a) displays the temperature dependence of the longitudinal resistivity (*ρ*_xx_). For electrical measurements, the sample was defined into a Hall-bar geometry (inset of Fig. 2(b)) to reduce mixing of the longitudinal and Hall resistances. A metal-insulator transition (MIT) is observed around transition temperature (*T*_M_) of 105 K, which is close to the reported value for bulk (120 K, Ref. 24). The absolute values of *ρ*_xx_ and the strength of the MIT sensitively depend on the growth conditions (data for typical films are shown in Fig. S4(a)), likely as a result of film-dependent Eu/Ir nonstoichiometry. Since analytical determination on the composition of thin films is challenging, we estimate the composition of films by comparing the resistivity ratio *ρ*_xx_(2 K)/*ρ*_xx_(300 K) with those of previously reported polycrystalline bulk data^25^. The result indicates that the films are Ir-rich by 1–4% (Fig. S4(b)). The reduction of *T*_M_ compared with the bulk may also be explained by the cation nonstoichiometry. Irrespective of quantitative variations in *ρ*_xx_, we qualitatively obtain the same magnetotransport properties discussed later for all the thin films. Hence, we focus on the most conducting film (sample 3 in Supplementary Fig. S4) in the structural and transport data presented. As also noted in Ref. 25 for polycrystalline bulk samples, the temperature dependence of Hall coefficient (*R*_H_) estimated by Hall measurement (see Methods) does not show significant anomaly across *T*_M_, in contrast to the sharp kink at MIT in *ρ*_xx_ as shown in Fig. 2(b). Although the origin of this behaviour remains controversial^26^,^27^, we simply refer to this transition as the MIT.

### Magnetoresistance

**Figure 3:**
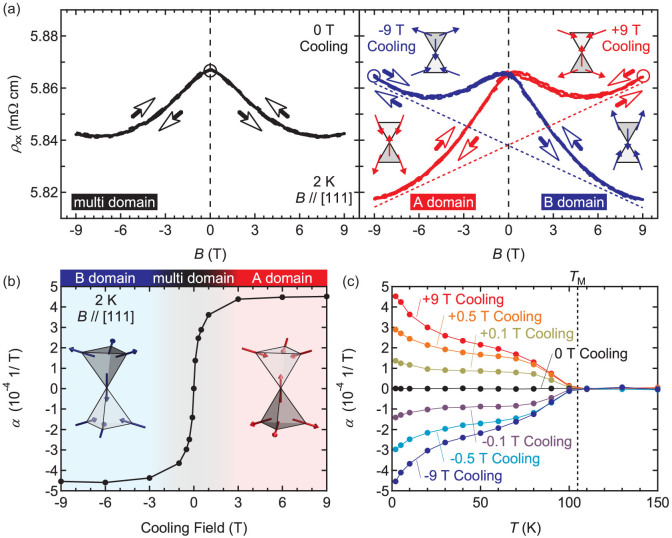
Linear magnetoresistance mediated by all-in-all-out spin structure. (a) Magnetic field dependence of longitudinal resistivity *ρ*_xx_ with *B/*/[111] at 2 K after zero field cooling (left panel) and ±9 T cooling (right panel). While the zero-field cooled data shows a symmetric response, *ρ*_xx_ after field cooling includes the linear terms indicated by dashed lines. Magnetic responses of Ir^4+^ magnetic moments corresponding to respective magnetic domain structures are schematically shown as insets, where spins are depicted as solid arrows and magnetic domains are symbolized by the pair of open and shaded triangles. The initial points and the direction of the magnetic field sweeps are indicated by open circles and open arrows, respectively, for each measurement. (b) Cooling field dependence of the linear magnetoresistance coefficient *α* at 2 K. *α* saturates above the cooling field of ±3 T, suggesting that magnetic domain be uniformly aligned to A-domain or B-domain. A multi-domain structure is formed by cooling in smaller magnetic fields. (c) Temperature dependence of *α* for representative cooling magnetic fields, showing that *α* appears only below the magnetic transition temperature *T*_M_.

Magnetotransport experiments provide versatile information reflecting the orbital motion and spin states of electrons, as exemplified by the spin liquid recently detected by the zero-field anomalous Hall effect in Pr_2_Ir_2_O_7_ (Ref. 28). Figure 3(a) shows the out-of-plane MR at 2 K. The MR for zero-field cooling (left panel) shows a symmetric shape with respect to zero field with a roughly 0.5% decrease in resistance at high magnetic field - a typical response in magnetic materials. The sample cooled with an applied magnetic field, however, unexpectedly exhibits an asymmetric term in MR in addition to the symmetric term (right panel). Although the symmetric term is found to be independent of cooling field (Fig. S5), the sign of the asymmetric term is changed by inverting the polarity of the cooling field. We emphasize that this asymmetric term is an intrinsic property of *ρ*_xx_ and is not caused by intermixing from Hall resistivity (*ρ*_yx_), as evidenced by the fact that the two-terminal MR shows qualitatively the same asymmetric term (Supplementary Fig. S6).

This observation appears peculiar as MR usually does not include an asymmetric term and is not dependent on the cooling field. Although magnetic metals or semiconductors show field asymmetric MR around zero field with a hysteretic behaviour due to a finite coercive field, it is symmetric when spins are flipped at high magnetic field. The MR observed in the data presented here can phenomenologically be understood by taking into account the all-in-all-out spin structure, based on the double exchange model as in the case of perovskite manganites. Here, the angle between the neighbouring spins dramatically affects the hopping probability, giving rise to MR^29^. For example, if A-domain is stabilized with cooling under +9 T, the spins on basal planes are canted towards the in-plane direction with positive magnetic field resulting in an increase in *ρ*_xx_ (top right inset). Conversely, a negative magnetic field tends to align those spins, giving rise to a lower *ρ*_xx_ (bottom left inset). The opposite response arises in the case of B-domain with −9 T field cooling. We have additionally checked the angle dependence of MR, which would reflect the crystallographic symmetry (Supplementary Figs. S9(a) and S9(b)). It turns out that the out-of-plane anisotropy is rather strong probably due to the structural shape anisotropy of the film and/or lattice distortion by epitaxial strain as is often the case of magnetic thin films (Supplementary Figs. S9(c) and S9(d)). Thus we utilize other methods for further verification as described in the following section.

### Temperature and cooling field dependence of the linear component in *ρ*_xx_

To further explore this idea, the same measurements are systematically performed as a function of temperature and cooling field (see Methods). Here, we define a parameter *α* to describe the sign and the magnitude of the asymmetric term as [*ρ*_xx_(*B*) − *ρ*_xx_(0)]/*ρ*_xx_(0) = (even term) + *αB*, since the odd terms equal to or higher than the third order are not significant (less than 1% of the linear term by fitting at 2 K). Figure 3(b) shows *α* as a function of cooling field at 2 K, indicating that *α* saturates above the cooling field of ±3 T. This bistability suggests that A-domain or B-domain may be selectively stabilized by applying a cooling field above ±3 T. The temperature dependence of *α* shown in Fig. 3(c) is also consistent with the above scenario as *α* disappears above *T*_M_. Although small hysteresis is observed around 60 K, the asymmetric term is clearly observed even in such a temperature region (Supplementary Fig. S7), indicating that one magnetic domain is dominant over the other.

### Hall measurements

**Figure 4:**
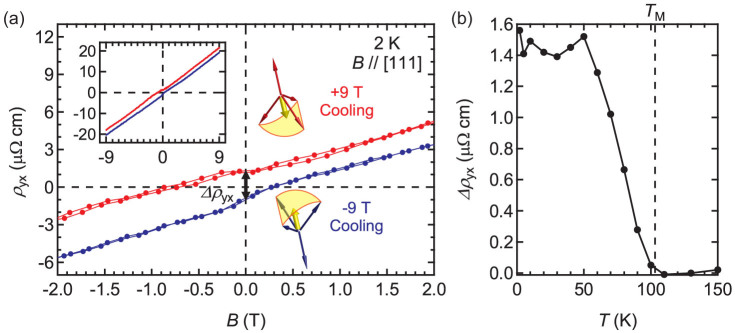
Anomalies in Hall resistivity *ρ*_yx_ originating from all-in-all-out spin structure. (a) Magnified view of magnetic field dependence of the Hall resistivity *ρ*_yx_ with *B/*/[111] at 2 K after ±9 T field cooling. *Δρ*_yx_ is defined as the difference of *ρ*_yx_ (0 T) between +9 T and −9 T field cooling. The scalar spin chirality induced by lattice distortion is schematically shown. The inset shows the data for the entire sweep range between ±9 T. (b) Temperature dependence of *Δρ*_yx_. The transition temperature *T*_M_ is indicted by a dashed line.

So far we have demonstrated that the MR contains a linear term reflecting the formation of a domain structure depending on the cooling field. We now show that the Hall resistance additionally captures the lattice distortion through the anomalous terms. *ρ*_yx_ is generally expressed as
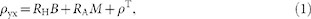
with *R*_H_ the ordinary Hall coefficient, *R*_A_ the Hall coefficient proportional to the macroscopic magnetization *M* as a result of spin-orbit interaction, and *ρ*^T^ the topological term induced by spin chirality *χ* = **S***_i_*·(**S***_j_* × **S***_k_*) (Ref. 30). Finite *M* and *χ* may remain in the presence of uniaxial lattice distortion induced by epitaxial strain, while they should be cancelled out in the case of ideal all-in-all-out spin structure with cubic symmetry. Figure 4(a) shows the raw data of the Hall measurement at 2 K for the cooling magnetic fields of ±9 T. In addition to the ordinary *B*-linear component common in both measurements, the Hall resistance clearly exhibits a non-zero value at 0 T, as measured by the difference in *ρ*_yx_ between the opposite cooling magnetic fields (Δ*ρ*_yx_). The overall linear term is reasonably assigned to the ordinary Hall effect (the first term in Eq. (1)), since no anomaly is present across *T*_M_ as shown in Fig. 2(b). We can therefore ascribe Δ*ρ*_yx_ to the sum of the second and third terms in Eq. (1) as Δ*ρ*_yx_ disappears above *T*_M_ (Fig. 4(b)).

## Discussion

As opposed to the standard field-symmetric MR typically observed in prevalent conductors, the magnetoresponse in the presence of all-in-all-out spin ordering is described in terms of a third rank tensor. Therefore, odd terms should characteristically be observed in response functions due to time-reversal symmetry breaking as proposed by Arima^31^, which is a clear fingerprint of the all-in-all-out spin ordering. Here, conduction electrons must be sensitive to the local spin structure of the compound owing to spin-orbit coupling, known as the Kondo lattice model. Hence, our observation substantiates that MR reflects not only the magnetic s*pin* structure but also the magnetic *domain* structure. From these considerations, the magnetic domain structure in Eu_2_Ir_2_O_7_ is robust against a magnetic field of at least 9 T below *T*_M_. This is in marked contrast to the case of Nd_2_Ir_2_O_7_ where A-domain and B-domain are thought to be switchable through the Ising character of Nd ions by sweeping magnetic field^32^. Therefore, it is plausible to attribute the robust domain structure to the *J* = 0 character of Eu^3+^.

The anomalies found in *ρ*_yx_ are consistent with the interpretation of MR data in which conducting carrier reflects the magnetic domain structure in another way, and additionally indicate that the lattice distortion actually induces macroscopic *M* and *χ*. An attempt to directly measure *M* by magnetrometer is not successful since *M* of this compound is still too small to compare with the signal from the substrate, yet *ρ*_yx_ is sufficiently sensitive to detect it without involving the substrate. In addition to Δ*ρ*_yx_, there exists a low-field nonlinear component (*ρ*_NL_), which is deduced by subtracting the linear term and the zero-field offset (Δ*ρ*_yx_) as shown in Fig. S8. Remarkably, *ρ*_NL_ shows sign inversion between the two domains. If we assume that *M* is proportional to *B* as in the case of the bulk^25^, *ρ*_NL_ may be therefore attributed to *ρ*^T^ possibly originating from spins at the surface or the interface. Confirming thickness dependence of magnetotransport properties would be useful comparison to clarify this issue. However, clear dependence is not obtained probably due to slight fluctuation of the stoichiometry from sample to sample as mentioned above, while the qualitative features of MR and Hall effect are maintained. Although this sign inversion in *ρ*_NL_ is an intriguing character of this system, the details remain to be investigated including the possibility of band crossing as in the case of EuTiO_3_ (Ref. 33) or SrRuO_3_ (Ref. 34).

In conclusion, we have fabricated single crystal Eu_2_Ir_2_O_7_ thin films via solid phase epitaxy and systematically explored the magnetotransport properties originating from the all-in-all-out spin order. The MR contains a linear field dependent term while the Hall resistance has zero field offset and anomalous bending, both of which inherently depend on the type of domain formed. Our findings indicate that the all-in-all-out spin structure is controllable by the polarity of the cooling field and once formed is robust below *T*_M_. The ability to realize this spin structure in thin film form will become the cornerstone to realizing WSM phases via tailored pyrochlore iridate heterostructures and/or utilizing electrostatic gating.

## Methods

The Eu_2_Ir_2_O_7_ (111) films were prepared on commercial YSZ (111) single crystal substrates by pulsed laser deposition. Phase-mixed ceramic targets fabricated by hot-press method at 950°C under 25 MPa pressure were used with a prescribed ratio of Eu/Ir = 1/3. The films were deposited at a substrate temperature of 500°C in an atmosphere of 100 mTorr Ar gas containing 1% O_2_. A KrF eximer laser (λ = 248 nm) was used for ablating the target, with a fluence and frequency of 6 J/cm^2^ and 10 Hz. The films were in an amorphous phase after the deposition, with the pure Eu_2_Ir_2_O_7_ phase appearing after annealing in an electrical muffle furnace at 1000°C for 1.5 hours in air.

The Hall bar structure was processed by conventional photolithography and Ar ion milling. Ohmic Ni (10 nm)/Au (50 nm) contacts were deposited by an electron-beam evaporator. Magnetotransport measurements were performed in a liquid He cryostat equipped with a 9 T superconducting magnet (PPMS, Quantum Design Co.). The ordinary Hall coefficient (*R*_H_) is calculated by linear term in Hall resistance above ±5 T region. Temperature dependence measurements for *α* (Fig. 3(c)) were carried out by the following sequence. Magnetic field was applied at 150 K along [111] direction of YSZ substrate. Then the samples were cooled from 150 K down to 2 K under the fixed magnetic field. At 2 K, magnetotransport was measured as follows. In the case of positive (negative) field cooling, magnetic field was first set to −9 T (+9 T), then returned to +9 T (−9 T), and finally back to the initial cooling field. For example, in the case of +0.5 T cooling, magnetic field was swept as +0.5 T →−9 T → +9 T →+0.5 T. After this measurement, the sample was warmed to the next measurement temperature with +0.5 T of magnetic field.

## Author Contributions

T.C.F. performed the sample fabrication, measurements, and analysis. Y.K. assisted with the sample fabrication, measurements, and analysis. M.U., A.T. and T.A. assisted with the planning and analysis. M.K. directed the project.

## Supplementary Material

Supplementary Information Supplementary information

## References

[b1] Murakami S., Nagaosa N. & Zhang S.-C. Spin-Hall insulator. Phys. Rev. Lett. 93, 156804 (2004). 15524922 10.1103/PhysRevLett.93.156804

[b2] Bernevig B. A., Hughes T. L. & Zhang S.-C. Quantum spin Hall effect and topological phase transition in HgTe quantum wells. Science 314, 1757–1761 (2006). 17170299 10.1126/science.1133734

[b3] König M., Wiedmann S., Brüne C. & Roth A. Quantum spin Hall insulator state in HgTe quantum wells. Science 318, 766–770 (2007). 17885096 10.1126/science.1148047

[b4] Hasan M. Z. & Kane C. L. Colloquium: Topological insulators. Rev. Mod. Phys. 82, 3045–3067 (2010).

[b5] Yan B. & Zhang S.-C. Topological materials. Rep. Prog. Phys. 75, 096501 (2012). 22907264 10.1088/0034-4885/75/9/096501

[b6] Qi X.-L. & Zhang S.-C. Topological insulators and superconductors. Rev. Mod. Phys. 83, 1057–1110 (2011).

[b7] Yu R. *et al.* Quantized anomalous Hall effect in magnetic topological insulators. Science 329, 61–64 (2010). 20522741 10.1126/science.1187485

[b8] Chang C.-Z. *et al.* Experimental observation of the quantum anomalous Hall effect in a magnetic topological insulator. Science 340, 167–170 (2013). 23493424 10.1126/science.1234414

[b9] Checkelsky J. G. *et al.* Trajectory of the anomalous Hall effect towards the quantized state in a ferromagnetic topological insulator. Nat. Phys. 10, 731–736 (2014).

[b10] Murakami S. Phase transition between the quantum spin Hall and insulator phases in 3D: emergence of a topological gapless phase. New J. Phys. 9, 356 (2007).

[b11] Murakami S. & Kuga S. Universal phase diagrams for the quantum spin Hall systems. Phys. Rev. B 78, 165313 (2008).

[b12] Burkov A. A. & Balents L. Weyl semimetal in a topological insulator multilayer. Phys. Rev. Lett. 107, 127205 (2011). 22026796 10.1103/PhysRevLett.107.127205

[b13] Halász G. & Balents L. Time-reversal invariant realization of the Weyl semimetal phase. Phys. Rev. B 035103, 1–9 (2012).

[b14] Guo H.-M. & Franz M. Three-dimensional topological insulators on the pyrochlore lattice. Phys. Rev. Lett. 103, 206805 (2009). 20366001 10.1103/PhysRevLett.103.206805

[b15] Wan X., Turner A. M., Vishwanath A. & Savrasov S. Y. Topological semimetal and Fermi-arc surface states in the electronic structure of pyrochlore iridates. Phys. Rev. B 83, 205101 (2011).

[b16] Yang K., Lu Y. & Ran Y. Quantum Hall effects in a Weyl semimetal: Possible application in pyrochlore iridates. Phys. Rev. B 84, 075129 (2011).

[b17] Wei H., Chao S. & Aji V. Excitonic Phases from Weyl semimetals. Phys. Rev. Lett. 109, 196403 (2012). 23215410 10.1103/PhysRevLett.109.196403

[b18] Subramanian M., Aravamudan G. & Subba Rao G. V. Oxide pyrochlores — A review. Prog. Solid State Chem. 15, 55–143 (1983).

[b19] Sagayama H. *et al.* Determination of long-range all-in-all-out ordering of Ir^4+^ moments in a pyrochlore iridate Eu_2_Ir_2_O_7_ by resonant x-ray diffraction. Phys. Rev. B 87, 100403 (2013).

[b20] Tomiyasu K. *et al.* Emergence of magnetic long-range order in frustrated pyrochlore Nd_2_Ir_2_O_7_ with metal–insulator transition. J. Phys. Soc. Jpn. 81, 034709 (2012).

[b21] Yamaji Y. & Imada M. Metallic interface emerging at magnetic domain wall of antiferromagnetic insulator: Fate of extinct Weyl electrons. Phys. Rev. X 4, 021035 (2014).

[b22] Cordfunke E. H. P. & Meyer G. The system iridium - oxygen I. Measurements on the volatile oxide of iridium. Recl. des Trav. Chim. des Pays-Bas 81, 495–504 (1962).

[b23] Kim B. *et al.* Novel *J*_eff_ = 1/2 Mott State Induced by Relativistic Spin-Orbit Coupling in Sr_2_IrO_4_. Phys. Rev. Lett. 101, 076402 (2008). 18764560 10.1103/PhysRevLett.101.076402

[b24] Matsuhira K., Wakeshima M., Hinatsu Y. & Takagi S. Metal–Insulator Transitions in Pyrochlore Oxides *Ln*_2_Ir_2_O_7_. J. Phys. Soc. Jpn. 80, 094701 (2011).

[b25] Ishikawa J. J., O'Farrell E. C. T. & Nakatsuji S. Continuous transition between antiferromagnetic insulator and paramagnetic metal in the pyrochlore iridate Eu_2_Ir_2_O_7_. Phys. Rev. B 85, 245109 (2012).

[b26] Mandrus D. *et al.* Continuous metal-insulator transition in the pyrochlore Cd_2_Os_2_O_7_. Phys. Rev. B 63, 195104 (2001).

[b27] Zhao S. *et al.* Magnetic transition, long-range order, and moment fluctuations in the pyrochlore iridate Eu_2_Ir_2_O_7_. Phys. Rev. B 83, 180402 (2011).

[b28] Machida Y., Nakatsuji S., Onoda S., Tayama T. & Sakakibara T. Time-reversal symmetry breaking and spontaneous Hall effect without magnetic dipole order. Nature 463, 210–213 (2010). 20010605 10.1038/nature08680

[b29] Ramirez A. P. Colossal magnetoresistance. J. Phys. Condens. Matter 9, 8171–8199 (1997).

[b30] Nagaosa N., Yu X. Z. & Tokura Y. Gauge fields in real and momentum spaces in magnets: monopoles and skyrmions. Philos. Trans. A. Math. Phys. Eng. Sci. 370, 5806–19 (2012). 23166382 10.1098/rsta.2011.0405

[b31] Arima T. Time-reversal symmetry breaking and consequent physical responses induced by all-in-all-out type magnetic order on the pyrochlore lattice. J. Phys. Soc. Jpn. 82, 1–4 (2013).

[b32] Ueda K. *et al.* Anomalous domain-wall conductance in pyrochlore-type Nd_2_Ir_2_O_7_ on the verge of the metal-insulator transition. Phys. Rev. B 89, 075127 (2014).

[b33] Takahashi K. S., Onoda M., Kawasaki M., Nagaosa N. & Tokura Y. Control of the anomalous Hall effect by doping in Eu_1−*x*_La*_x_*TiO_3_ thin films. Phys. Rev. Lett. 103, 057204 (2009). 19792531 10.1103/PhysRevLett.103.057204

[b34] Fang Z. *et al.* The anomalous Hall effect and magnetic monopoles in momentum space. Science 302, 92–95 (2003). 14526076 10.1126/science.1089408

