# Supplementary material for: Odd-parity magnetoresistance in pyrochlore iridate thin films with broken time-reversal symmetry
Source: Sci Rep. 2015 May 11;5:9711. doi: 10.1038/srep09711 (PMC4426595; doi:10.1038/srep09711)
Supplement: Supplementary Information [file srep09711-s1.doc]

Odd-parity magnetoresistance in pyrochlore iridate thin films with broken time-reversal symmetry

T. C. Fujita^1^, Y. Kozuka^1,*^, M. Uchida^1^, A. Tsukazaki^1,2,3^, T. Arima^4,5^, M. Kawasaki^1,5^.

^1^Department of Applied Physics and Quantum-Phase Electronics Center (QPEC), University of Tokyo, Tokyo 113-8656, Japan.

^2^Institute for Materials Research, Tohoku University, Sendai 980-8577, Japan.

^3^PRESTO, Japan Science and Technology Agency (JST), Tokyo 102-0075, Japan.

^4^Department of Advanced Materials Science, University of Tokyo, Kashiwa 277-8561, Japan.

^5^RIKEN Center for Emergent Matter Science (CEMS), Wako 351-0198, Japan.

**Supplementary Materials**

**Growth phase diagram**

Single phase Eu_2_Ir_2_O_7_ films were prepared by pulsed laser deposition. Although pyrochlore phase has never been formed in as-grown films, we succeeded in obtaining single crystalline phase by solid state epitaxy as well as optimizing laser energy, composition of the target, and annealing conditions. Substrate temperature and oxygen partial pressure (*P*_O2_) during deposition were controlled as indicated in the phase diagram shown in the top panel of Fig. S1(a). The diagram can be divided into four regions based on the results of XRD as shown in Figs. S1(b) – S1(e) for representative films. In the (b) region, only Eu_2_O_3_ peaks appeared. Compositional analysis revealed the absence of Ir, possibly due to the evaporation of Ir containing spices such as IrO_3_ with a very high vapour pressure^22^. In the (c) region, in addition to the peaks of Eu_2_O_3_, those of metallic Ir appeared, indicating excessively reductive conditions. In the (d) region, both Ir and Eu are in the amorphous phase as detected by energy dispersive X-ray spectroscopy. In the (e) region, single phase Eu_2_Ir_2_O_7_ appeared by annealing in an electrical furnace at 1000 °C for 1.5 hours in air. Samples within the (d) region but outside of the (e) region shown by a box contained some impurity phases such as Ir metal and/or polycrystalline Eu_2_O_3_ even after annealing under the same conditions. Broken lines indicate the phase coexistence conditions for respective pairs of oxidation states of iridium, which were determined by electrochemical calculation.


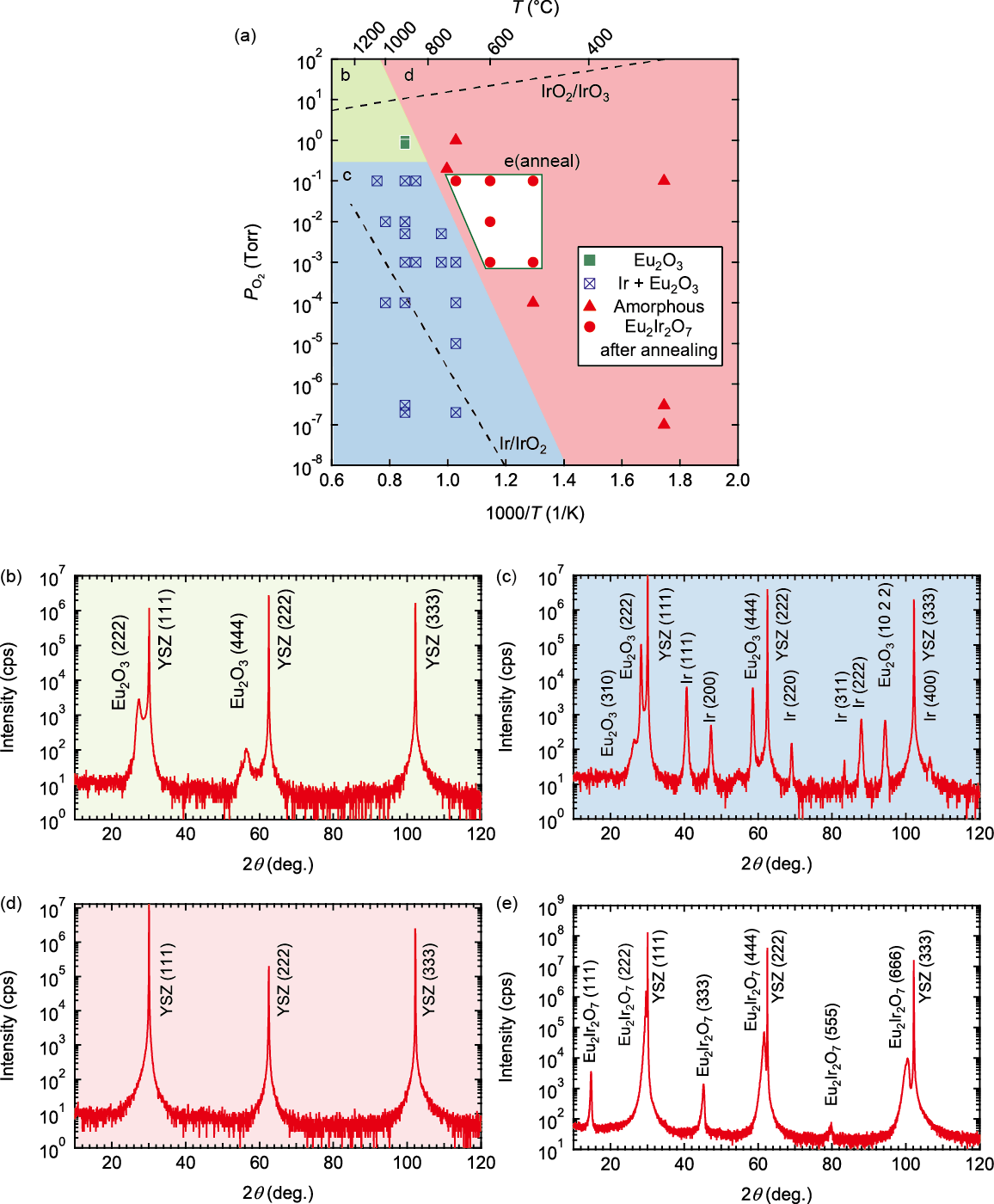
**Figure S1 | A phase diagram of Eu_2_Ir_2_O_7_ films grown on YSZ (111) substrate.** (a) The growth phase diagram as functions of *P*_O2_ and temperature, classified into the following four regions based on X-ray diffraction. (b) Ir evaporates and only Eu remains in the form of Eu_2_O_3_. (c) Ir is crystallized and Eu forms polycrystalline Eu_2_O_3_. (d) Both Ir and Eu are in the amorphous phase. (e) Eu_2_Ir_2_O_7_ phase appears after annealing although it is amorphous in the as-grown state. Broken lines indicate the phase coexistence conditions for respective pair of iridium oxides.

**X-ray diffraction**

Additional XRD results are shown in Fig. S2. The rocking curve around Eu_2_Ir_2_O_7_ (222) peak is shown in Fig. S2(a). The full width at half maximum (FWHM) is 0.085°. Azimuthal (*φ*) scan around Eu_2_Ir_2_O_7_ (662) in Fig. S2(b) shows three-fold symmetry, which is the same as that of the YSZ substrate, indicating that our films contain neither misoriented domain nor stacking faults. Different peak intensities at different *φ* are due to the slight misalignment of the sample.


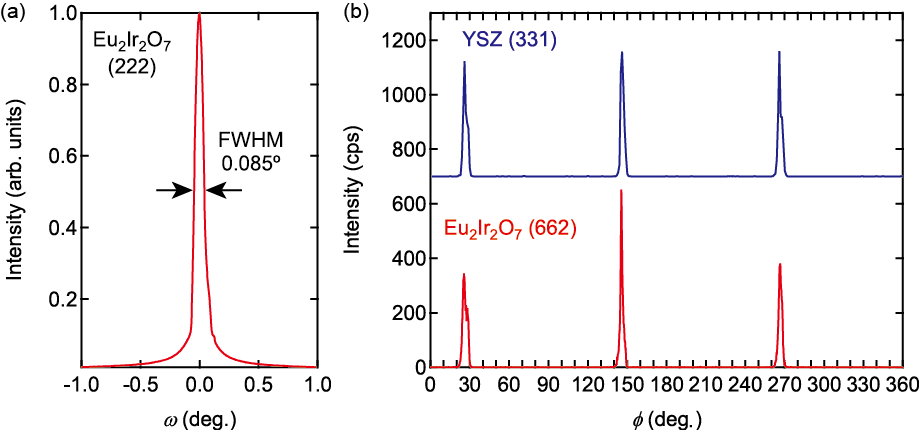


**Figure S2 | Rocking curve and azimuthal scan.** (a) Rocking curve around Eu_2_Ir_2_O_7_ (222) peak. (b) Azimuthal scan around Eu_2_Ir_2_O_7_ (662) peak. The Eu_2_Ir_2_O_7_ film shows three-fold symmetry.

**Atomic force microscopy**

Atomic force microscope (AFM) image of the sample before and after annealing in the region of 2 μm square are shown in Fig. S3. The route mean square roughness (RMS) is order of 1 nm for both before and after annealing. Although surface morphology is quite different between before and after annealing, RMS is almost the same.


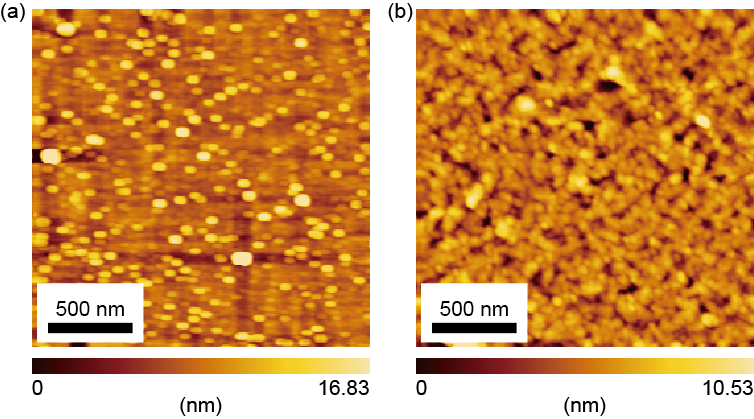


**Figure S3 | AFM image before and after annealing.** (a) before and (b) after annealing.

**Temperature dependence of resistivity**

Figure S4 shows temperature dependence of the longitudinal resistivity (*ρ*_xx_) and *ρ*_xx_(2 K)/*ρ*_xx_(300 K) for typical Eu_2_Ir_2_O_7_ thin films. In the main part of the paper, the data obtained in No. 3 sample are discussed. The large variation of *ρ*_xx_ and strength in MIT are thought to originate from the cation off-stoichiometry that was controlled by varying Ir/Eu composition in the target; Ir/Eu = 1 and 1.5 for samples 1 and 2, respectively. Other growth conditions were the same for all the samples. From a simple comparison with bulk polycrystalline samples in the previous report^25^, our samples are off-stoichiometric by about 1 – 4 % Ir-rich.


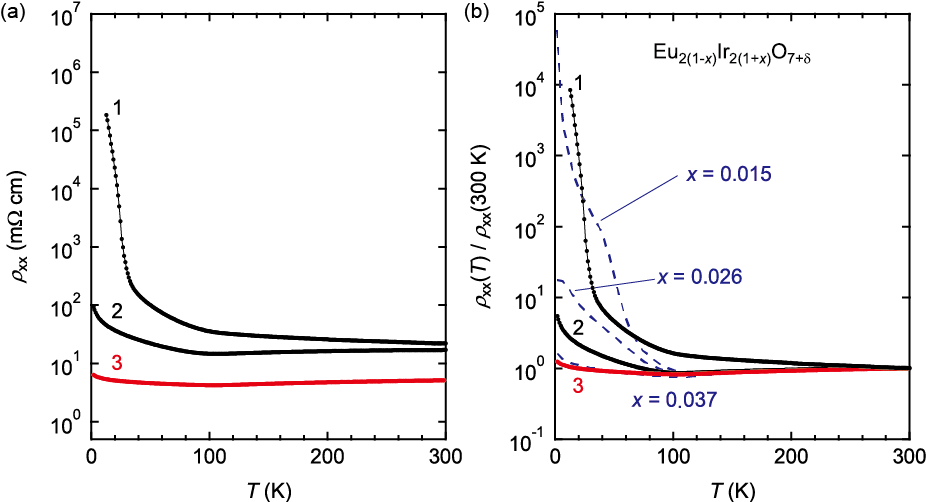


**Figure S4 | Temperature dependence of *ρ*_xx_ and *ρ*_xx_(*T*)/*ρ*_xx_(300 K).** (a) The data for three representative samples are shown. The sample No. 3 (highlighted by red colour) is discussed in the main part. The data for bulk polycrystalline samples in the previous paper^25^ are also shown in (b) as dashed curves for comparison after normalized by the value at 300 K. Chemical composition is expressed in the formula Eu_2(1-_*_x_*)Ir_2(1+_*_x_*)O_7+δ_, where *δ* is nearly zero (Ref. 25).

**(Anti-)symmetrization of magnetoresistance**

Figure S5(a) displays the symmetric and asymmetric terms in *ρ*_xx_ extracted from the data in Fig. 3(a). The symmetric terms completely coincide for three different cooling conditions, and can be interpreted as ordinary magnetoresistance possibly originating from delocalization by orbital effect. In contrast, asymmetric terms only appear in field cooling data as shown in Fig. S5(b). This asymmetric term is dominated by a linear term, the polarity of which is switched with inverting the cooling field direction. (A)symmetric terms are calculated by a conventional (anti-)symmetrization defined as follows:

$\begin{matrix} \rho_{xx}sym=\frac{\rho_{xx}(B)+\rho_{xx}(-B)}{2}, \\ \rho_{xx}asym=\frac{\rho_{xx}(B)-\rho_{xx}(-B)}{2}. \end{matrix}$ (S1)

**
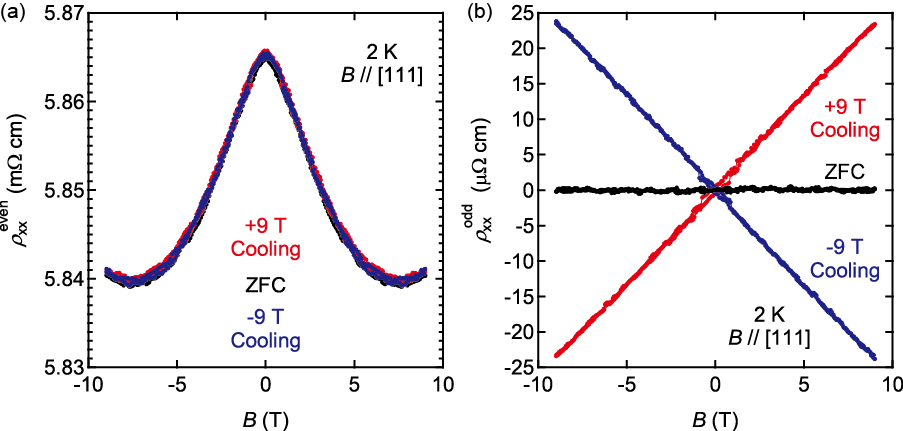
**

**Figure S5 | Symmetrized and anti-symmetrized magnetoresistance.** (a) Symmetric and (b) asymmetric terms are extracted from the data shown in Fig. 3(a).

**Two-terminal magnetoresistance**

Figure S6 shows two-terminal longitudinal resistivity (*ρ*_xx2_) as a function of magnetic field (*B*) to exclude any contribution from *ρ*_yx_. A linear term is clearly observed in magnetoresistance and the polarity is inverted, depending on cooling field direction. This result rules out the possibility of mixing with *ρ*_yx_ as a source of linear term in *ρ*_xx_.


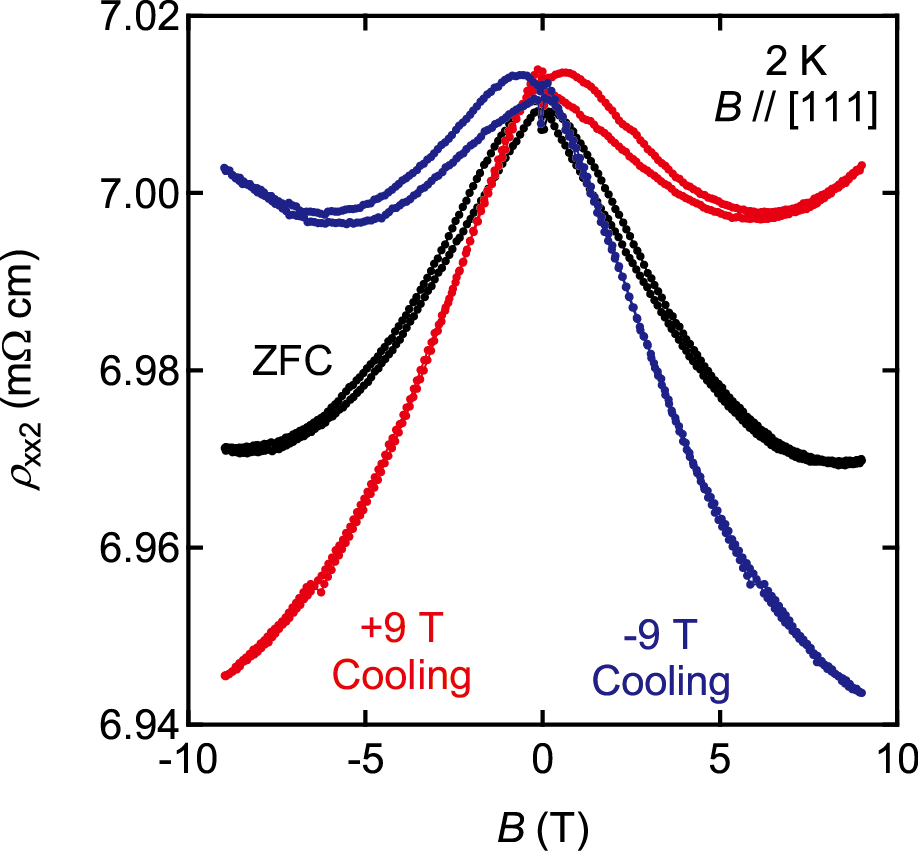


**Figure S6 | Two-terminal resistivity.** Raw data of the two-terminal longitudinal resistivity (*ρ*_xx2_) at 2 K for strained film after cooling under 0 T (ZFC) and $\pm$9 T.

**(Anti-)symmetrizated magnetoresistance at various temperatures**

In Fig. S7 we show the symmetric (Fig. S7(a)) and asymmetric (Fig. S7(b)) term of *ρ*_xx_ defined by Eq. (S1) for several temperatures under different cooling magnetic fields (0 T and $\pm$9 T). At lower temperatures below 50 K, symmetric term shows almost the same behaviour independent of cooling field and do not have hysteresis. With increasing temperature to 70 K, weak but finite hysteresis is observed, and it disappears again above *T*_M_ (105 K). In contrast, the asymmetric term highly depends on cooling field, having exactly opposite sign between +9 T and $-$9 T, and almost zero for 0 T cooling, and such behaviour is independent of measuring temperature below *T*_M_. From these results, we can conclude that (i) the magnetic domain structure is completely fixed at lower temperatures, (ii) above around 60 K, other ferromagnetic ordering may emerge due to the sweep magnetic field, (iii) no magnetic order exists above *T*_M_. The coercive field can be extremely high of the order of ~ 100 T in the case of antiferromagnetic frustrated systems, but it is not clear for the present system at the moment.

The temperature of 60 K is consistent with a temperature at which Δ*ρ*_yx_ shown in Fig. 4(b) is saturated whereas *α* shown in Fig. 3(b) is not. One possible origin of this difference is considered to be (i) the development of other magnetic order or (ii) the existence of coupling parameter between carriers and magnetic moments. Particularly for (ii), both carrier conduction and magnetism originate from 5*d* electron of Ir in this system, which would further enhance such coupling. In order to verify our consideration determining exact magnetic structure for this system is inevitable and this remains as a future work.


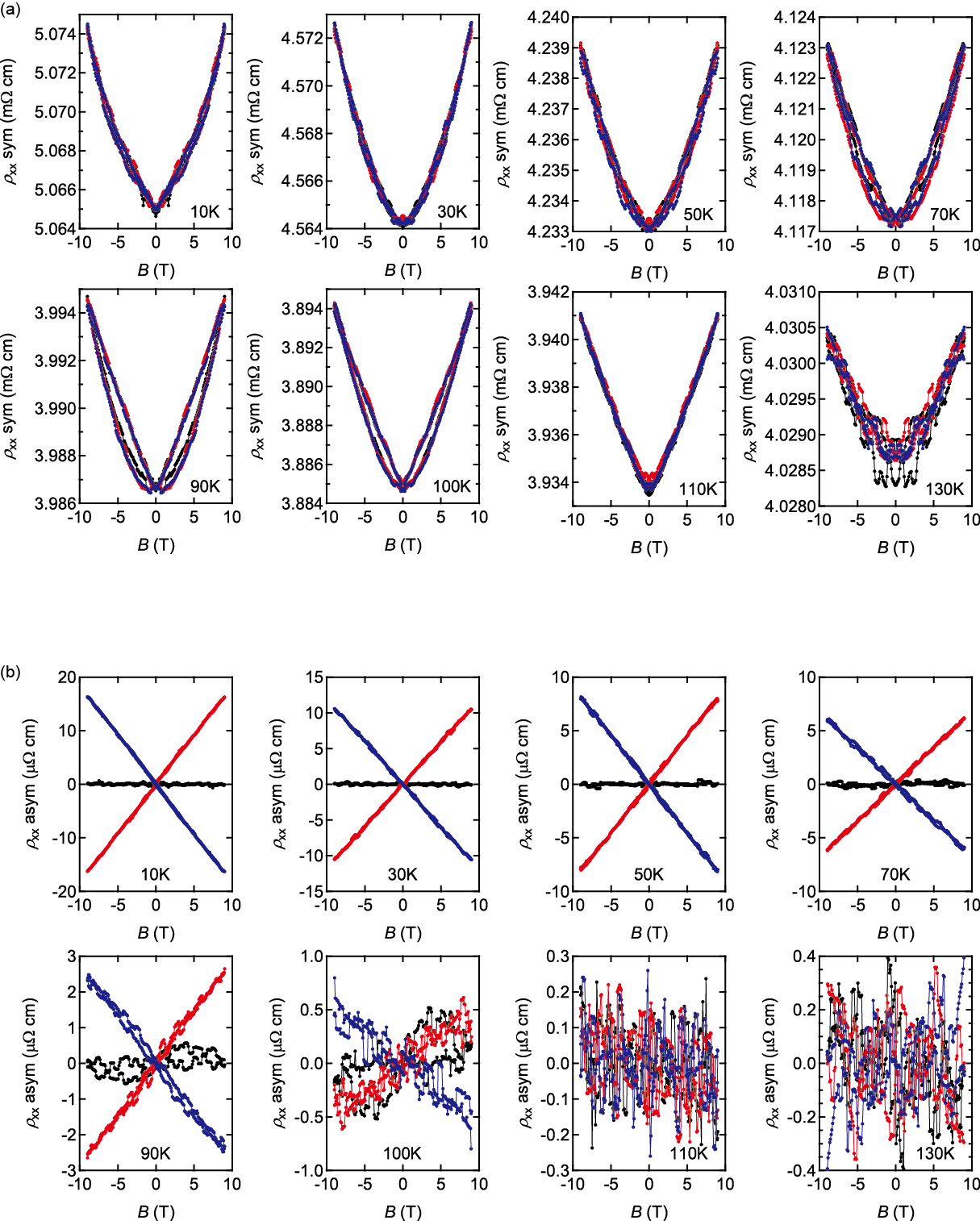


**Figure S7| Temperature dependence of symmetrized and anti-symmetrized magnetoresistance.** (a) Symmetric and (b) asymmetric terms in magnetoresistance shown in Fig. 3(a) after cooling under 0 T (ZFC) and $\pm$9 T.

**Nonlinear terms in Hall resistivity**

Figure S8(a) shows the field-asymmetric terms (*ρ*_yx_ asym) in *ρ*_yx_ from the data in Fig. 4(a), which is calculated by deducing Δ*ρ*_yx_. Here, we note that these terms exhibit bending around 0 T, the directions of which are opposite depending on the cooling field, namely magnetic domain structure. By subtracting *B*-linear ordinary Hall term from *ρ*_yx_, we can extract non-linear components (*ρ*_NL_) of *ρ*_yx_ (Fig. S8(b)). Although the origin is still unclear as mentioned in main text, *ρ*_NL_ should be intrinsic property in this system reflecting the magnetic structure because the sign change in the anomalous Hall effect does not occur in general by extrinsic sources such as magnetic impurities. One of the possible reasons for these observations is that band crossing near the Fermi level generates Berry curvature which induces fictitious magnetic flux as reported in the case of EuTiO_3_ (Ref. 33) or SrRuO_3_ (Ref. 34), and this would be validated by carrier level control with using field-effect transistor technique for our thin films. Another possibility is the existence of the isolated magnetic domains at the interface/surface of the film as pointed in main text, because inversion symmetry is spontaneously broken there and net magnetic moment could remain. In this case, *ρ*_NL_ is affected by the termination (kagome plane or triangular plane) and dimensionality (should be enhanced in thinner film) of the film, and further refinements of on growth technique is inevitable.


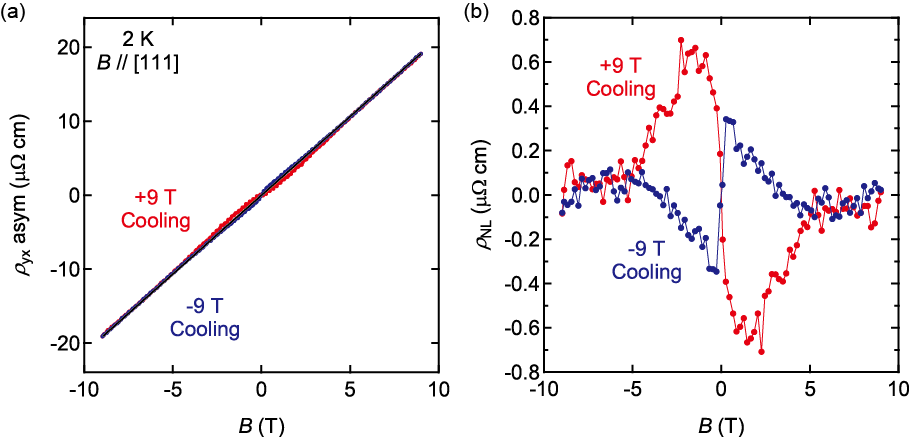


**Figure S8 | Non-linear term in Hall resistivity.** (a) Asymmetric and (b) non-linear components (*ρ*_NL_) in Hall resistivity at 2 K for strained film after $\pm$9 T of magnetic field cooling from the data in Fig. 4(a).

**Angle dependence of magnetoresistance**

We have measured angle dependence of the magnetoresistance after cooling the sample under +9 T along the field direction of [111]. As shown in Fig. S9(a), the sample is rotated about [10] axis that is current flow direction. Crystallographycally, [111] is equivalent to [1], and [] is equivalent to [11] with taking into account all-in-all-out magnetic ordering (Fig. S9(b)). In Fig. S9(c), we present the MR data at 2 K when the field is rotated away from [111] direction after +9 T (−9 T) cooling along [111]. As opposed to the crystallographic symmetry, MR shows cosine-like angle dependence (Fig. S9(d)). This result suggests that the growth direction is a unique axis for the MR among <111> directions because the coefficient for linear MR (*α*) is not the same between [111] and [1]. From this observation, we speculate that the shape anisotropy of the film and/or lattice distortion by epitaxial strain strongly affects the magnetotransport in our films as is often the case of magnetic thin films.


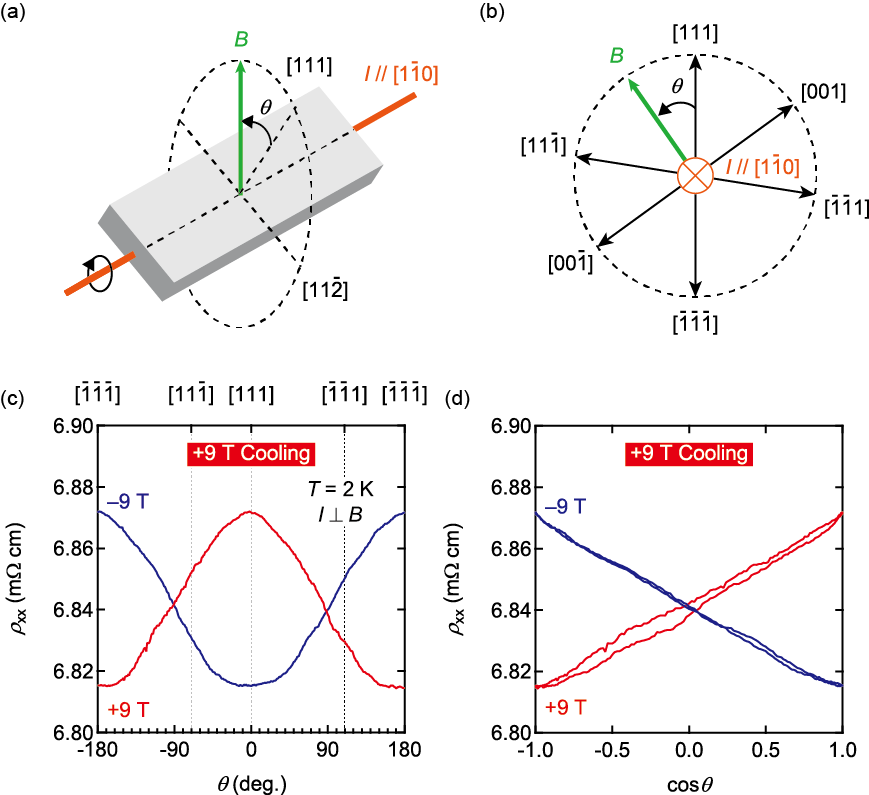


**Figure S9 | Magnetoresistance under the rotated field.** (a) Schematic diagram of the sample configuration. (b) Relative position between the representative crystal directions and the [111]-direction. (c) Angle dependence of *ρ*_xx_(+9 T) (red) and *ρ*_xx_($-$9 T) (blue) after +9 T magnetic field cooling. (d) The same data as panel (c) as a function of cos*θ*. The current *I* flows along [10] direction and sample is rotated about the same axis.
